# Drug elucidation: invertebrate genetics sheds new light on the molecular targets of CNS drugs

**DOI:** 10.3389/fphar.2014.00177

**Published:** 2014-07-28

**Authors:** Donard S. Dwyer, Eric Aamodt, Bruce Cohen, Edgar A. Buttner

**Affiliations:** ^1^Department of Psychiatry–Department of Pharmacology, Toxicology and Neuroscience, Louisiana State University Health Sciences Center-ShreveportShreveport, LA, USA; ^2^Department of Biochemistry and Molecular Biology, Louisiana State University Health Sciences Center-ShreveportShreveport, LA, USA; ^3^Department of Psychiatry, Harvard Medical SchoolBoston, MA, USA; ^4^Mailman Research Center, McLean HospitalBelmont, MA, USA; ^5^Department of Neurology–Department of Psychiatry, McLean Hospital, Harvard Medical SchoolBelmont, MA, USA

**Keywords:** anesthetics, antidepressants, antipsychotics, drug discovery, ethanol, *Caenorhabditis elegans*

## Abstract

Many important drugs approved to treat common human diseases were discovered by serendipity, without a firm understanding of their modes of action. As a result, the side effects and interactions of these medications are often unpredictable, and there is limited guidance for improving the design of next-generation drugs. Here, we review the innovative use of simple model organisms, especially *Caenorhabditis elegans*, to gain fresh insights into the complex biological effects of approved CNS medications. Whereas drug discovery involves the identification of new drug targets and lead compounds/biologics, and drug development spans preclinical testing to FDA approval, drug elucidation refers to the process of understanding the mechanisms of action of marketed drugs by studying their novel effects in model organisms. Drug elucidation studies have revealed new pathways affected by antipsychotic drugs, e.g., the insulin signaling pathway, a trace amine receptor and a nicotinic acetylcholine receptor. Similarly, novel targets of antidepressant drugs and lithium have been identified in *C. elegans*, including lipid-binding/transport proteins and the SGK-1 signaling pathway, respectively. Elucidation of the mode of action of anesthetic agents has shown that anesthesia can involve mitochondrial targets, leak currents, and gap junctions. The general approach reviewed in this article has advanced our knowledge about important drugs for CNS disorders and can guide future drug discovery efforts.

## INTRODUCTION

**FIGURE 1:**
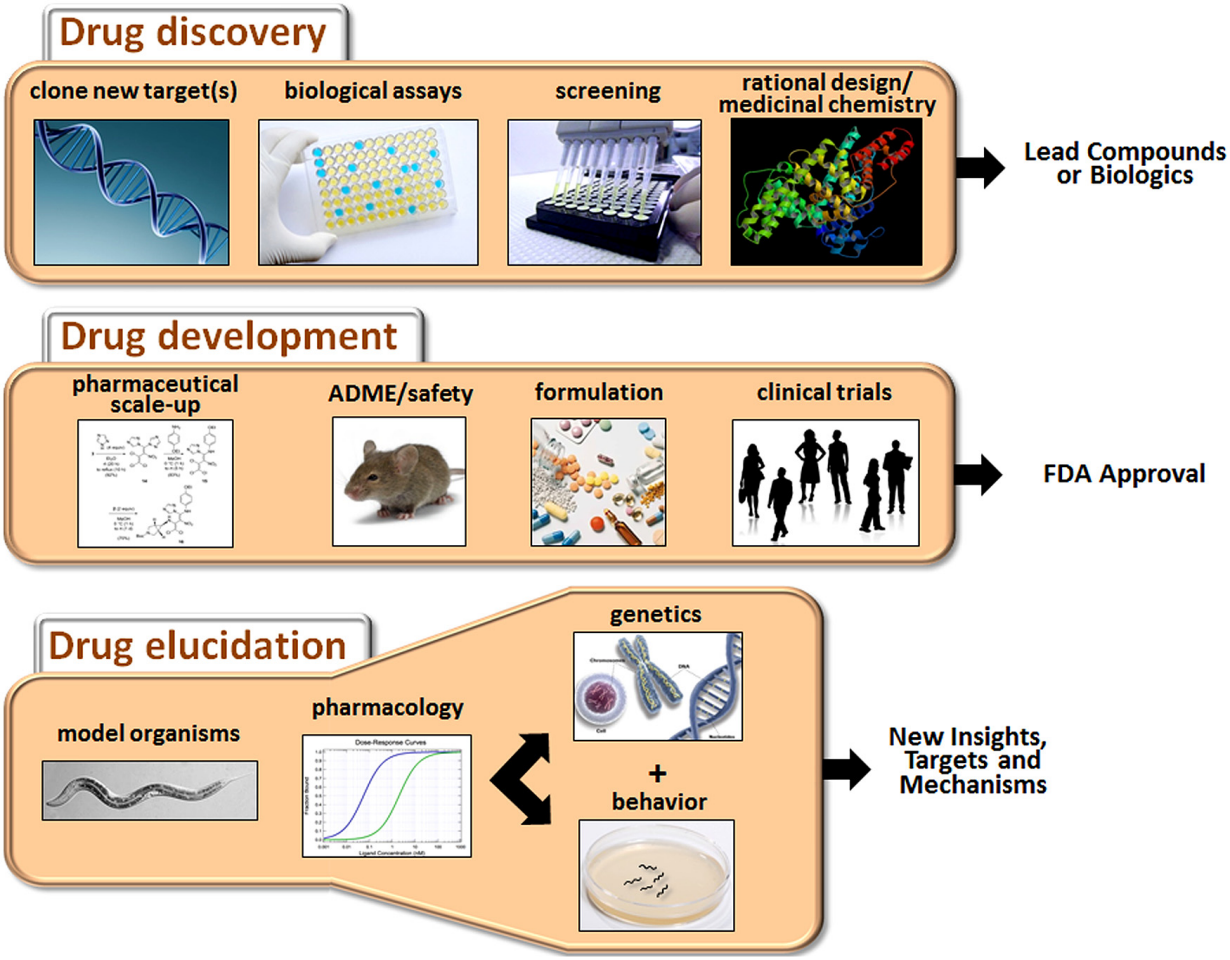
**Major activities of drug discovery, drug development, and drug elucidation.** The discovery process begins with the identification of appropriate targets or desired activities in biological assays (e.g., neuroprotection or cytotoxicity). Active compounds are then identified by screening or *de novo* design, depending on knowledge about the target. The activity of the lead compounds can be improved by rational drug design (based on pharmacophore analysis or co-crystal structures with the receptor) and medicinal chemistry. During the drug development stage, the chemistry is scaled up, and pharmaceutical quality batches are produced for *in vivo* testing. Drug candidates are evaluated for ADME properties and toxicity in animals. Candidates clearing these hurdles are suitably formulated and assessed in clinical trials. Success in these trials will determine whether a drug receives FDA approval. Drug elucidation is achieved by ongoing evaluation of marketed drugs in animals, including model organisms such as *C. elegans*. Through a combination of pharmacology, genetics and behavioral assessment, novel targets can be identified, and/or new insights into therapeutic mechanisms and side effects can be obtained. This information can then guide the next round of drug discovery and so on.

The process of developing new drugs is historically divided into two phases that reflect the different goals and tasks of this complex effort (**Figure 1**). Drug discovery is the initial phase characterized by a search for appropriate targets or effects and identification of small molecules or biologics that selectively modulate those targets (Hughes et al., 2011). A target is typically selected on the basis of accumulated evidence linking it mechanistically to a disease, for example, mutation of the transmembrane conductance regulator in cystic fibrosis. However, for many CNS disorders, the identity of the targets directly mediating the disorder (e.g., defective gene products or disease-causing variants) or even the extended pathways that could be targeted for ameliorative effects are not known with any certainty. The second phase of the process is drug development, which is focused on promising drug candidates identified in discovery-stage research. The main goals of this phase are to scale up chemistry and formulation, demonstrate safety in animals, and ultimately, perform clinical trials to assess tolerability and therapeutic benefits in patients (Venkatesh and Lipper, 2000).

This review article will focus on *drug elucidation* as a third phase of drug discovery/development aimed at more thorough characterization of the biological activities of FDA-approved medications. We will begin with a broad discussion of the challenges faced in developing new drugs for CNS disorders, and why it is, therefore, important to fully characterize medications already on the market to treat these conditions. To illustrate this point, several examples will be provided where the innovative use of *Caenorhabditis elegans* has revealed novel findings or complemented other work on the molecular actions of antipsychotics, antidepressants, anesthetics, and other CNS drugs. Although the main focus is on *C. elegans* as a model system, we will also occasionally highlight the use of *Drosophila* and cell-based systems to gain insights into these drug classes. Because the literature on these other systems is extensive, we refer the reader to selected reviews that give a flavor for recent developments (Chiu and Chuang, 2010; Quesseveur et al., 2013; Siebel et al., 2014; Urs et al., 2014). Finally, we will outline how drug elucidation can guide future research directions and possibly reveal new indications for existing drugs.

## CHALLENGES IN THE DEVELOPMENT OF NEW AND IMPROVED CNS DRUGS

Over the past several years, there have been unfortunate late-stage failures of drugs in clinical trials of agents believed to be specifically targeted to pathological mechanisms of various psychiatric and neurological illnesses. This includes failure of a glutamate 2/3 receptor agonist (LY2140023) for schizophrenia (Stauffer et al., 2013), preladenant, an adenosine 2A receptor antagonist aimed at Parkinson’s disease (Clinicaltrials. gov, 2013), and semagacestat, a γ-secretase inhibitor for the treatment of Alzheimer’s disease (Doody et al., 2013). There are many possible reasons for these failures, including inadequate knowledge about suitable targets and their relevant biology, redundancy in signaling pathways, pharmacokinetic issues that cast doubt on whether adequate therapeutic levels of drug were achieved, off-target effects, the potential irreversibility of late-stage dysfunction in these disorders, and placebo effects in control subjects. As a result of these and other setbacks, most major pharmaceutical companies have dramatically reduced or curtailed in-house research efforts to develop drugs for CNS diseases. This decision is based largely on the realization that our knowledge about the causes and pathogenesis of most of these disorders is inadequate.

Regarding the role of genetic factors in disease causation, mutation of a single gene or even a few genes rarely explains the risk profile or complex symptoms and patterns of functional decline in patients with various CNS diseases. Instead, these conditions, including schizophrenia and Parkinson’s disease, appear to result from the deleterious effects of concurrent alterations in multiple interacting genes, together with environmental effects. This means that, to be effective, either a drug must impact several proteins or functional pathways, or a cocktail of several drugs must be used. Even when contributory genes have been identified [e.g., disrupted in schizophrenia-1 (DISC1); Millar et al., 2001], CACNA1C in several psychiatric disorders (Bhat et al., 2012), or α-synuclein in Parkinson’s disease (Nussbaum and Polymeropoulos, 1997), their precise roles in pathogenesis remain unclear. To address serious gaps in our knowledge about the role of genetic factors, researchers are now attempting to deconvolute CNS disorders into simpler components, known as endophenotypes (Gottesman and Gould, 2003), which are the manifestations of genotypes associated with specific aspects of a disease, e.g., dysconnectivity of regional neural activity in schizophrenia (Karbasforoushan and Woodward, 2012).

Despite our limited understanding of disease causation, drugs have successfully been developed to treat various psychiatric and neurological conditions. For instance, chlorpromazine was developed to treat schizophrenia, lithium to treat bipolar disorder, and riluzole for amyotrophic lateral sclerosis (ALS). Unfortunately, many of these drugs produce only modest benefits compared to placebo, and none is considered a cure.

Currently available CNS drugs have, nevertheless, provided useful clues about the diseases they target based on their putative mechanisms of action. The pharmacological effects of antipsychotics and Parkinson’s drugs implicate altered dopaminergic function in schizophrenia (Carlsson, 1974) and Parkinson’s disease (Cotzias et al., 1969). While dopamine is only part of the story in these cases, the initial insights provide stepping stones for developing more comprehensive theories about the relevant mechanisms of illness. Riluzole is the only FDA-approved drug for the treatment of ALS (Cheah et al., 2010). Preclinical studies suggest that it works, in part, by modulating glutamatergic neurotransmission, and decreasing excitotoxicity, thereby reducing motorneuron loss (Doble, 1996; Cheah et al., 2010). Similarly, the positive effects of cholinesterase inhibitors on memory function in Alzheimer’s patients support the idea of defective cholinergic function in this disease (Greenwald and Davis, 1983). However, in each case, the drugs may act downstream of the underlying pathological factors, rather than at the source. Also, there is still much to learn about the biological effects of the most efficacious CNS drugs, and how they can be improved. That is, the full effects of existing drugs are still unknown, and defining those effects potentially offers a rich source of new insights into disease mechanisms, and guidance for the development of next-generation therapeutics.

## PROPERTIES OF DRUGS AND OFF-TARGET EFFECTS

Before discussing how drug elucidation has begun to reveal unexpected targets and novel biological effects, it is worth remarking on the properties of the “perfect” CNS drug. Ideally, it would be orally bioavailable, readily cross the blood brain barrier, show high selectivity for its target, and produce no serious side effects. To attain this gold standard, candidate molecules undergo a rigorous selection process biased toward drug-like properties (e.g., Lipinski’s rule of five; Lipinski et al., 1997; Brüstle et al., 2002). Affinity and selectivity are guiding forces in drug development, yet they can also be impediments to truly rational drug design. High affinity is achieved by introducing chemical reactivity into lead compounds. However, an increase in reactivity to improve affinity may also increase the likelihood of off-target binding, especially in the absence of 3-D structural information to maximize complementarity at the binding site.

Selectivity is created by exploring different structural frameworks and arranging reactive groups (pharmacophores) so that binding is limited to the target receptor and possibly a few related receptors (Dror et al., 2004; Kawasaki and Freire, 2011). However, at therapeutic concentrations, many “selective” drugs will bind to additional lower affinity sites. Moreover, selectivity is established by measuring binding to closely related receptors, for example, additional G protein-coupled receptors (GPCR). However, screening across a wider range of target classes is also conducted, with a particular focus on targets known to have safety liability, such as the cardiac hERG channel. The production of active metabolites, with different binding properties, further complicates drug development.

It is interesting to consider whether the therapeutic effects of a drug are due mainly to its interaction with the primary target, which elicits very specific responses, or to interactions with a wider spectrum of proteins and broader biological activity. Antipsychotic drugs (APD) have multiple molecular mechanisms (e.g., see Cohen and Zubenko, 1985; Cohen and Lipinski, 1986). Moreover, Roth et al. (2004) and others (Ma et al., 2006) have argued that “dirty” drugs, with multiple effects, may actually be needed for the treatment of schizophrenia and major depression. Antipsychotics treat delusions and hallucinations in schizophrenia and bipolar disorder, but they are also used off-label to treat a wide variety of other neuropsychiatric conditions. Dopamine D_2_ receptor blockade is a shared feature of these widely used drugs, but it does not appear to be their sole mechanism of action (Grunder et al., 2009; Meltzer, 2013). Other targets likely to be relevant for the therapeutic effects of antipsychotics include cholinergic receptors (Ibrahim and Tamminga, 2011), glutamate and serotonin receptors (Fribourg et al., 2011; Fell et al., 2012; de Bartolomeis et al., 2013), and α-1 noradrenergic receptors (Cohen and Lipinski, 1986; Ma et al., 2006). These examples are not meant to be exhaustive, but are listed here to illustrate the point that antipsychotics are multi-target drugs (Roth et al., 2004) with additional molecular mechanisms yet to be elucidated.

The presence of reactive substituents in drugs, non-selective drug binding, drug accumulation in tissues or cells, and unanticipated actions of drug metabolites all contribute to the most serious consequence of off-target effects, namely, adverse events due to drug treatment. Some of the adverse events are predictable based on the pharmacological profile of the drug. Oftentimes though, the side effects are not explained by the known pharmacology, and reveal novel, unsuspected drug targets. This new knowledge can then be factored into the development of next-generation drugs with less liability for causing harmful effects. Therefore, it is important to have a comprehensive knowledge about the various targets of established medications to avoid side effects and to determine the key activities desired in future drugs. In the following sections, we describe how drug elucidation in model organisms has provided valuable insights into APDs, antidepressants, anesthetics and other CNS drugs, and the disorders that they treat.

## DRUG ELUCIDATION: NEW INSIGHTS INTO ANTIPSYCHOTIC DRUGS

**FIGURE 2:**
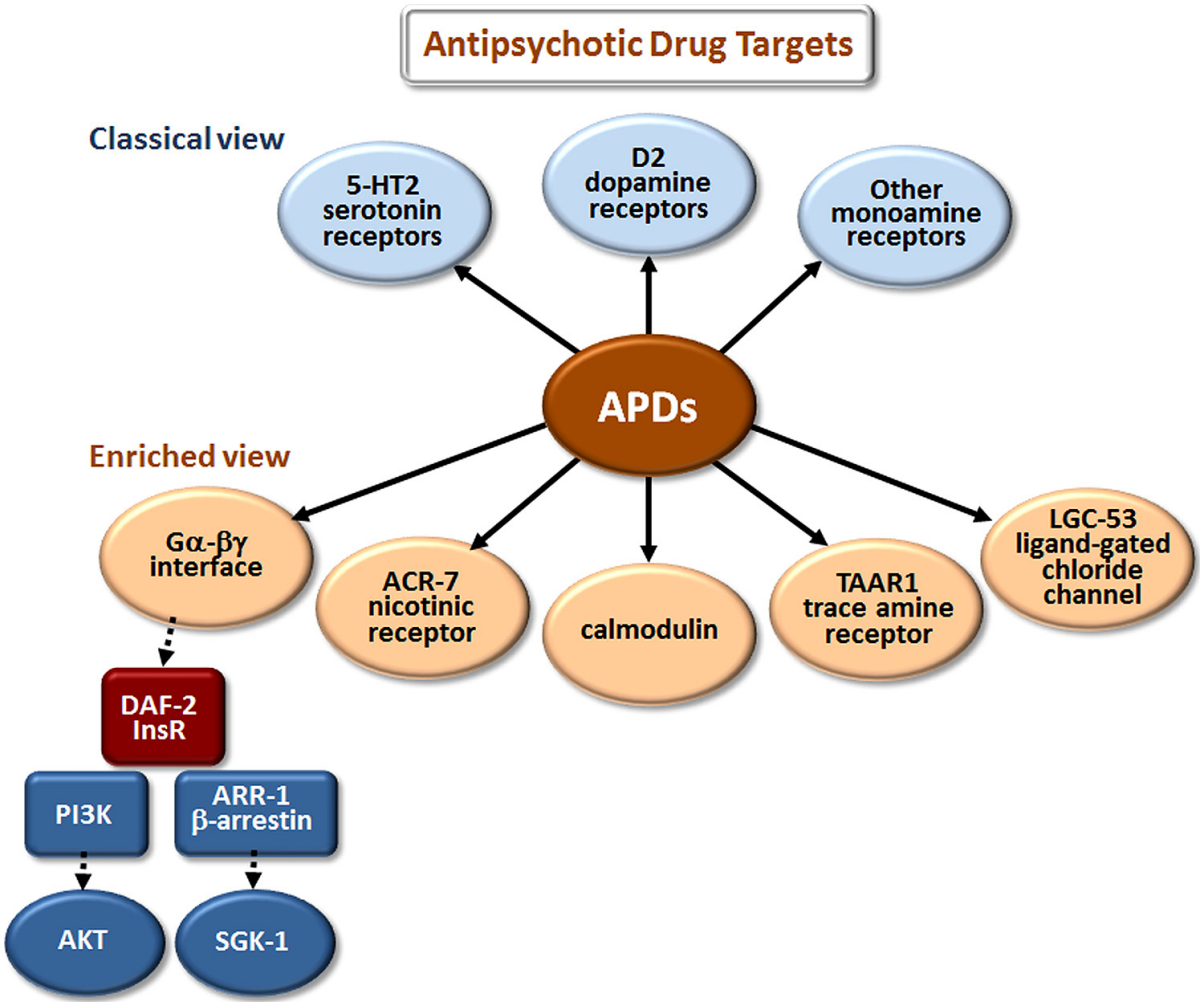
**Elucidation of additional targets of antipsychotic drugs (APDs).** The classical view refers to drug targets identified during the original development of first- and second-generation APDs. The enriched view depicts new targets identified in studies of these drugs in model organisms, primarily *C. elegans*. The drugs appear to bind directly (as indicated by solid arrows) to the first layer of targets, and have important secondary (red targets) and tertiary (blue targets) effects that are indirect (dashed arrows). Although the first-layer targets are depicted in parallel in **Figures 2–4**, they may actually operate in series, or as components of more extensive pathways. Gα-βγ denotes the G protein α and βγ subunits associated with G protein-coupled receptors (GPCRs). DAF-2 is the *C. elegans* insulin/IGF-1 receptor (InsR); PI3K is phosphatidylinositol 3-kinase; AKT and SGK-1 (serum- and glucocorticoid-inducible kinase-1) are serine/threonine kinases.

The classical view of APD action has focused on monoamine and, especially, dopamine and serotonin receptor antagonism (**Figure 2**). However, these are not the only sites where APDs have direct or indirect effects. Drug elucidation studies in *C. elegans* recently implicated the growth factor receptor DAF-2 and the ligand-gated ion channels (LGICs) ACR-7 and LGC-53 as APD targets (Karmacharya et al., 2009; Ringstad et al., 2009; Weeks et al., 2010; Saur et al., 2013). DAF-2 is orthologous to the mammalian insulin receptor, while ACR-7 is orthologous to a mammalian nicotinic acetylcholine receptor (nAChR) α subunit. LGC-53 is a tyramine-gated chloride channel, but whether LGC-53 has a mammalian ortholog remains unknown. APD effects in *C. elegans* were also shown to require signaling by trace amines, such as tyramine, a finding that was extended to mammals (Karmacharya et al., 2011), though relevance to humans remains untested.

Work from two independent groups showed that APDs activate the insulin signaling pathway (ISP) in *C. elegans*. First, Karmacharya et al. (2009) demonstrated that mutations in the insulin receptor gene *daf-2* and in a downstream effector, the phosphatidylinositol 3-kinase (PI3K) gene *age-1*, suppress larval arrest induced by the atypical APD clozapine. Clozapine also increased expression of an *age-1::GFP* reporter construct. Activation of the ISP is expected to cause cytoplasmic localization of the fork head transcription factor FOXO/DAF-16. Consistent with this expectation, clozapine produced cytoplasmic localization of DAF-16::GFP in arrested L1 larvae, whereas DAF-16::GFP was nuclear localized in L1 larvae arrested due to starvation or high temperature. Subsequently, Weeks et al. (2010) showed that all major classes of APDs increased signaling through the DAF-2/AGE-1/AKT-1,2 pathway, as demonstrated by a decrease in nuclear accumulation of DAF-16::GFP in starved *C. elegans*. The same group had previously shown that atypical APDs increase Akt phosphorylation in cultured mammalian neurons (Lu et al., 2004; Lu and Dwyer, 2005), findings consistent with earlier suggestions (Dwyer et al., 2003) and human genetic studies implicating dysfunction of the PI3K/AKT pathway in schizophrenia (Emamian et al., 2004). The *C. elegans* results extended the mammalian data by revealing specific mechanisms through which APDs induce Akt phosphorylation and through which APDs may thereby compensate for dysfunction of the PI3K/AKT pathway in schizophrenia. The results may prove relevant not only for understanding the fundamental pathogenetics of schizophrenia and the therapeutic mechanisms of action of APDs but also for identifying mechanisms underlying the toxic effects of APDs, such as metabolic syndrome. For example, Gubert et al. (2013) showed that the atypical antipsychotic ziprasidone altered lipid metabolism in *C. elegans* in a DAF-16-dependent manner.

Clozapine is the most effective APD for treatment-refractory schizophrenia, but the molecular basis of its unique therapeutic efficacy is not well understood (Meltzer, 2013). Cell-based studies have identified additional targets of clozapine, including T-type calcium channels (Choi and Rhim, 2010). Interestingly, drug elucidation studies in *C. elegans* revealed that clozapine differs from other APDs with respect to ISP activation. Clozapine’s effects required two components of the pathway that other APDs did not, the β-arrestin scaffolding protein ARR-1 and the serum- and glucocorticoid-inducible kinase SGK-1 (Weeks et al., 2010, 2011). Thus, clozapine may activate PI3K/AGE-1 and SGK-1 via β-arrestin/ARR-1 and may also act in parallel through PI3K/AGE-1 and PDK-1 to activate AKT-1,2. Of possible relevance to these observations, recent studies in *C. elegans* revealed that AKT-1 and SGK-1 affect lifespan, stress resistance, and DAF-16 activity in very different ways (Chen et al., 2013). For example, AKT-1 shortened lifespan, while SGK-1 promoted longevity in a DAF-16-dependent manner. AKT-1 reduced stress resistance, while SGK-1 promoted resistance to oxidative stress and ultraviolet radiation. AKT-1 promoted cytoplasmic localization of DAF-16, but SGK-1 did not. Importantly, effects of *sgk-1* mutations on DAF-16 target gene expression indicated that SGK-1 controlled DAF-16 targets through mechanisms that were distinct from those of AKT-1 (Chen et al., 2013). These results raise the possibility that the downstream consequences of ISP activation by clozapine may be quite different from that of other APDs, possibly contributing to the differential therapeutic effects of clozapine vis-à-vis other APDs.

ISP activation is not the only clozapine-specific effect to emerge from APD studies in *C. elegans*. Karmacharya et al. (2011) showed that clozapine stimulated egg-laying, an effect not seen with the typical antipsychotic haloperidol or the atypical antipsychotic olanzapine. A candidate gene screen revealed that clozapine-induced egg-laying required the gene *tdc-1*, responsible for tyramine biosynthesis. The generalizability of these findings to mammals was explored using trace amine-associated receptor 1 (TAAR1) knockout mice. Prepulse inhibition (PPI) of acoustic startle is used to identify APDs that predict therapeutic benefit in patients, and clozapine increased PPI in wild-type mice. This increase was abrogated in TAAR1 knockout mice, suggesting a role for TAAR1 in clozapine-induced PPI enhancement (Karmacharya et al., 2011).

While the discoveries of APD-induced ISP activation and APD-induced trace amine signaling in *C. elegans* arose from candidate gene screening, the power of invertebrate genetics to elucidate fundamentally new drug targets lies ultimately in the ability to conduct unbiased genetic screens for previously undocumented targets. Recently, taking this unbiased approach, Saur et al. (2013) reported a genome-wide RNA interference (RNAi) screen in *C. elegans* for new APD targets. The screening strategy took advantage of the developmental delay induced by APDs in this organism (Donohoe et al., 2006; Karmacharya et al., 2009), a phenotype thought to arise in part from APD-induced inhibition of pharyngeal pumping (Donohoe et al., 2009; Saur et al., 2013). Specifically, a genome-wide feeding RNAi screen was performed for *S*uppressors of *C*lozapine-induced *L*arval *A*rrest (*scla* genes). The primary screen tested 19,968 wells, representing ∼70% of currently annotated *C. elegans* genes, followed by subsequent testing of primary screen positives in triplicate. The approach yielded 40 candidate suppressors, a number of which were then validated using knockout mutants, including the α-like nAChR subunit *acr-7*. Expression of a translational *acr-7::GFP* construct in the *acr-7* knockout partially rescued suppression of both clozapine-induced developmental delay and clozapine-induced inhibition of pharyngeal pumping. These clozapine-induced phenotypes were phenocopied by nAChR agonists and blocked by nAChR antagonists. Taken as a whole, the results suggested that clozapine activates the ACR-7 receptor, a finding consistent with mammalian studies implicating nAChRs in the pathophysiology of schizophrenia (Harrison and Weinberger, 2005; Martin and Freedman, 2007). No other APDs have been shown to activate nAChRs, although α7-nAChR agonists are currently being tested as treatments for psychosis (Jones et al., 2012). Thus, α-like nAChR signaling constitutes a mechanism through which clozapine, and possibly other APDs, may produce their therapeutic or toxic effects in human patients.

ACR-7 is homologous to a variety of human α-like nAChRs, but the identity of the true ACR-7 ortholog is unknown. Mammals have at least 17 different nAChR subunits, and these nAChRs may assemble in a variety of functional combinations. Effects of APDs on the spectrum of nAChR subunit combinations have not been tested. Therefore, identifying the mammalian ortholog of ACR-7 could resolve the important question of whether APDs can activate one or more of these receptors. Detailed characterization of other suppressors from the genome-wide RNAi screen of Saur et al. (2013) is being conducted, as well.

A second LGIC, LGC-53, has emerged as a novel APD target from studies in *C. elegans*. The discovery originated from studies of mutant genes in animals defective for *M*odulation *O*f locomotion *D*efective (*mod* genes). First, Ranganathan et al. (2000) showed that the gene *mod-1* encodes a novel kind of serotonin receptor, a serotonin-gated chloride channel. Ringstad et al. (2009) then identified 26 presumptive Cys-loop family ion channels highly similar to MOD-1 and expressed them in oocytes to test for receptor activity. Three genes were found to encode biogenic amine-activated ion channels, and one of these, LGC-53, was activated with highest efficacy by dopamine and was antagonized with IC_50_’s in the range of 20–60 μM for the APDs haloperidol and risperidone. APDs can reach concentrations in tissues where they are concentrated (e.g., brain and fat) that are 20–30-fold higher than in serum (Tsuneizumi et al., 1992; Kornhuber et al., 1999). These results raise the interesting possibility that the therapeutic and toxic effects of APDs may involve inhibition of not only G protein-coupled dopamine receptors but also dopamine-gated ion channels. As with ACR-7, the next step in the LGC-53 project will be to identify a human counterpart.

The studies reviewed here underscore several important points regarding the mechanisms of APD action. First, the classical view that APDs regulate dopaminergic and serotonergic signaling is likely incomplete. Rather, APDs appear to interact with a variety of signaling pathways, some of which are novel. Unbiased genetic screens in animal models offer the possibility of identifying such novel pathways. Second, dissecting these pathways may elucidate targets that account for differences in the therapeutic efficacy of APDs. The role of SGK-1 in clozapine’s modulation of the ISP is a potential case in point. Third, while genetic screens may open a window onto new drug targets, follow-up studies of mammalian orthologs of targets such as ACR-7 and LGC-53 are essential.

## ANTIDEPRESSANT DRUGS

Antidepressant drugs (ADs) are among the most prescribed class of medications for adults between the ages of 20 and 59 and are used to treat several emotional, behavioral, and neurological problems (Gu et al., 2010). These include depression, anxiety, obsessive–compulsive disorder, eating disorders, and both chronic and neuropathic pain. Less commonly, doctors use these drugs to treat other conditions including attention-deficit hyperactivity disorder, post-traumatic stress disorder, premenstrual dysphoria, dysmenorrhea, migraines, sleep disorders, snoring and substance abuse.

**FIGURE 3:**
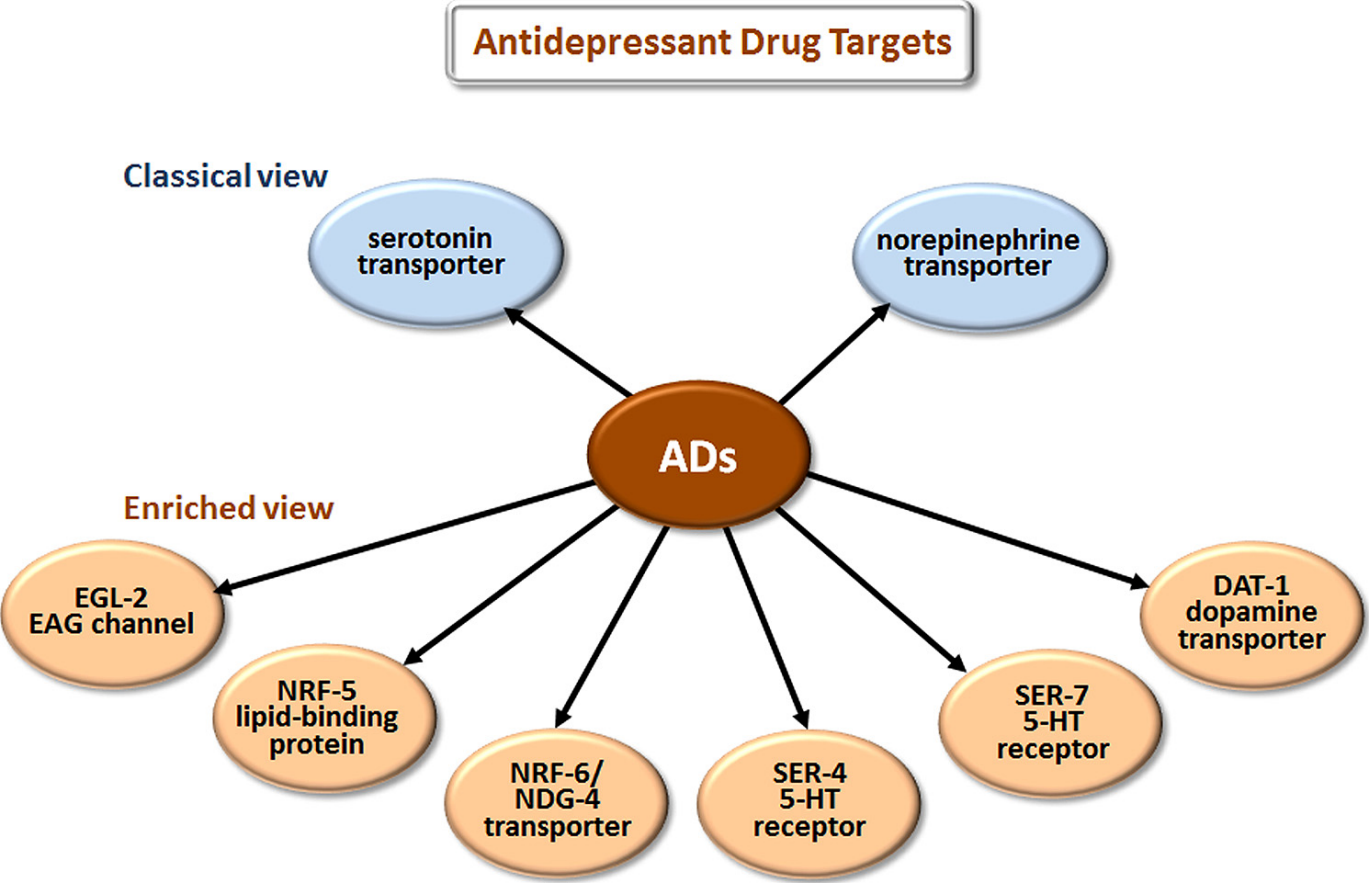
**Novel targets of antidepressant drugs (ADs).** According to the classical view, ADs work by inhibiting neurotransmitter reuptake via transporters of serotonin and norepinephrine. Drug elucidation has revealed that these drugs also bind to EAG K^+^ channels, lipid-binding proteins, additional transporters, and directly to particular serotonin receptors.

The most prescribed classes of AD are the selective serotonin reuptake inhibitors (SSRIs), serotonin-norepinephrine reuptake inhibitors (SNRIs), and the older tricyclic antidepressants (TCAs). The classical view (**Figure 3**) is that these drugs improve mood by blocking the reuptake of serotonin (5-HT) and/or norepinephrine at the synaptic cleft and thus increase signaling by these neurotransmitters.

ADs are effective in treating depression, but many patients respond incompletely, and ADs can have serious side effects. While recent blinded studies indicate that antidepressants or psychotherapies alone are only modestly better at symptom reduction than active intervention controls (Khan et al., 2012), the benefits of ADs to those with more severe symptoms are better established. The combination of psychotherapies and antidepressants may provide greater benefit (Khan et al., 2012). Common side effects of SSRIs and SNRIs include agitation during initiation of treatment, restlessness, dry mouth, blurred vision, headache, sedation, and elevated blood pressure. The list of side effects that are rare or for which the incidence is unknown is extensive and includes several that are very serious such as convulsions and suicide (Coupland et al., 2011; PubMed Health, 2013a). TCAs also have a very long list of possible side effects, especially autonomic effects, and several are again very serious (PubMed Health, 2013b). A clearer understanding of the systems affected by these drugs should help researchers identify more effective ADs with fewer side effects.

Antidepressants do more than block uptake of monoamine neurotransmitters. Studies with mammalian cells and mouse brain slices in culture have shown that SSRIs and TCAs bind tightly to several 5-HT receptor subtypes and act as either antagonists or agonists depending on the receptor bound (Ni and Miledi, 1997; Kroeze and Roth, 1998; Eisensamer et al., 2003). Mutations in *C. elegans* that either eliminate 5-HT, or knock out the 5-HT reuptake transporter, allowed several groups to show that the SSRI fluoxetine and the TCA imipramine can influence behavior independent of 5-HT and 5-HT reuptake (Weinshenker et al., 1995; Choy and Thomas, 1999; Sze et al., 2000; Ranganathan et al., 2001; Dempsey et al., 2005; Kullyev et al., 2010).

Studies in *C. elegans* have also elucidated several new antidepressant targets (**Figure 3**). EGL-2, the *C. elegans* homolog of ether-a-go-go, is a voltage-gated K^+^ channel required for egg-laying, muscle activation, defecation, mechanosensation, and chemosensation (Trent et al., 1983; Weinshenker et al., 1999; Wes and Bargmann, 2001). Activating K^+^ channels can lower cellular excitability, and inhibiting K^+^ channels can increase excitability. Imipramine inhibits EGL-2 K^+^ currents and currents from the mouse EGL-2 homolog mEAG (Weinshenker et al., 1999) and may thereby increase cellular excitability, which may explain some cardiac side effects. NRF-5 is a lipid-binding protein related to mammalian cholesterol-ester-binding proteins. Choy et al. (2006) identified the *nrf-5* and *nrf-6* genes in a screen for mutations that confer resistance to a fluoxetine-induced nose contraction, an effect that is independent of 5-HT reuptake. In the same genetic pathway as *nrf-5* are two genes that encode 12-pass transmembrane proteins, *nrf-6* and *ndg-4*. Loss-of-function mutations in each of these three genes reduce the fluoxetine-induced nose contraction and result in pale eggs, presumably due to yolk and lipid insufficiency. Together, NRF-5, NRF-6, and NDG-4 appear to be involved in transporting fluoxetine from the gut to its sites of action.

Fluoxetine, imipramine, and 5-HT all stimulate egg-laying in *C. elegans*. Deletion of SER-4, the *C. elegans* ortholog of the 5-HT1 receptor, strongly reduces egg-laying stimulated by imipramine while leaving the egg-laying response to both 5-HT and fluoxetine intact (Dempsey et al., 2005). SER-7 is an ortholog of mammalian 5-HT7 GPCRs. Kullyev et al. (2010) found that fluoxetine binds directly to SER-7, and *ser-7* loss-of-function mutants are resistant to fluoxetine-induced paralysis, a phenotype that is independent of 5-HT reuptake. DAT-1 is a *C. elegans* dopamine reuptake transporter. Very low imipramine concentrations (*K*_i_ = 1 nm) block dopamine reuptake activity of DAT-1 in both transiently transfected HeLa cells (Jayanthi et al., 1998) and in *C. elegans* cells in primary culture (Carvelli et al., 2004). Tricyclic ADs generally have limited affinity for human DATs (Buck and Amara, 1995). A novel way in which *Drosophila* has aided drug elucidation is by providing structural insights into nortriptyline binding to a monoamine transporter. Penmatsa et al. (2013) solved the co-crystal structure of the *Drosophila* dopamine transporter together with nortriptyline. This information should assist structure-based drug design.

Kullyev et al. (2010) also provided evidence for two additional aspects of fluoxetine action through genetic analyses in *C. elegans*. First, they found that fluoxetine regulates acetylcholine, GABA, and glutamate neurotransmission in the locomotory circuit independent of the sole 5-HT reuptake transporter, MOD-5. Second, they found *C. elegans* neurons that contain, but do not synthesize, 5-HT. These cells obtain all of their 5-HT through uptake by MOD-5. If neurons that obtain all of their 5-HT through uptake also exist in humans, antidepressant treatment might eliminate 5-HT in specific subpopulations of neurons as well as increase presynaptic 5-HT.

Cell-based studies showed that ADs modulate growth factor expression in neuroblastoma cells (Henkel et al., 2008). These findings add to the list of evidence that implicates defective neuronal plasticity in major depression and restored plasticity or outgrowth as therapeutic. Moreover, this work addresses the important issue of why ADs take several weeks to produce clinical benefits. Of course, binding to the targets mentioned above, including classical targets such as monoamine transporters, may elicit adaptive changes in neurons that require several weeks to effect a beneficial outcome. Together, these studies identify additional antidepressant targets and effects that may be important for either the clinical action of these drugs or their side effects.

## ANESTHETICS AND ALCOHOL

**FIGURE 4:**
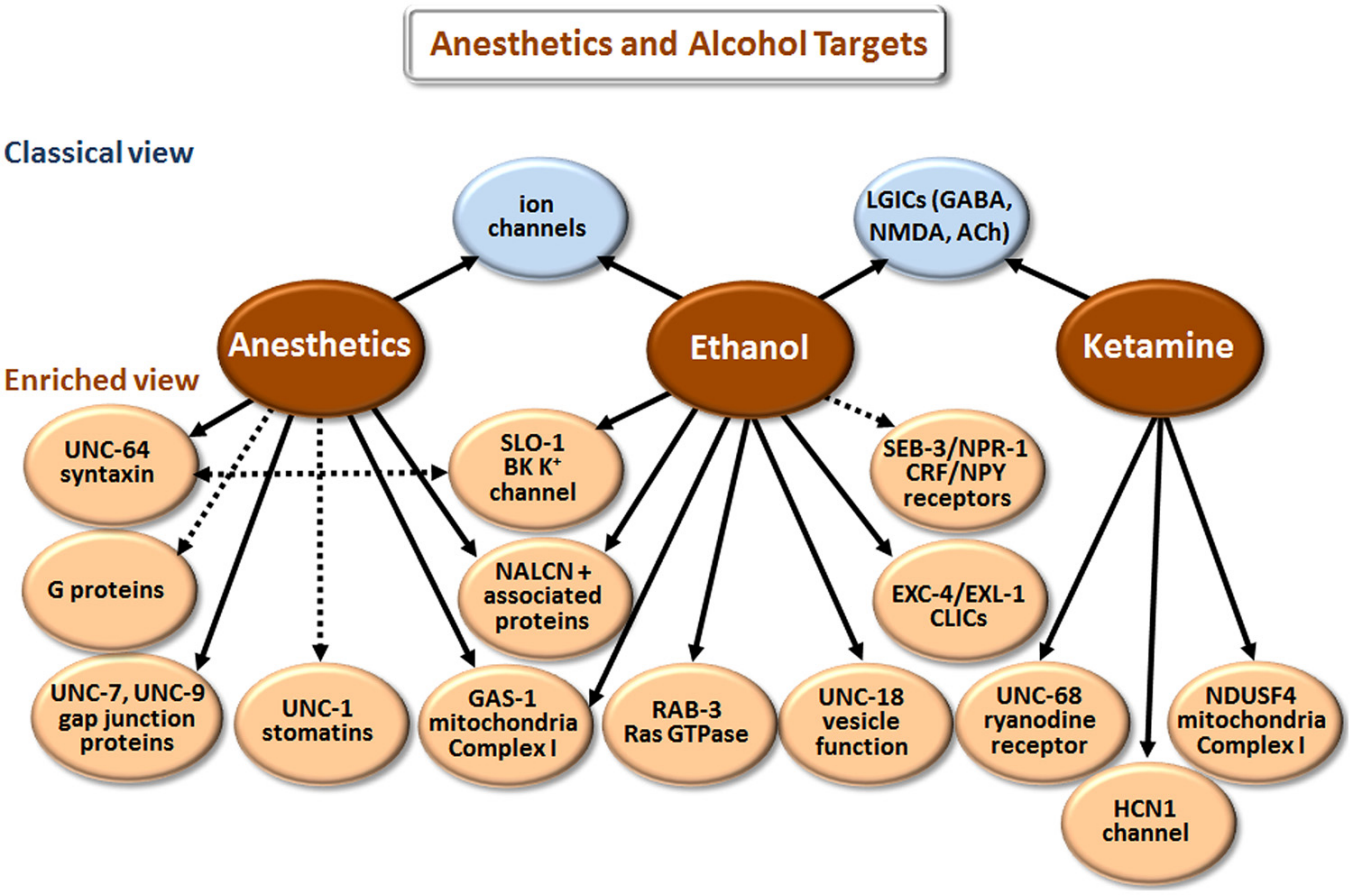
**Targets of anesthetics and ethanol identified in model organisms.** The classical view summarizes the main targets that have been implicated in man and mice/rats, or in electrophysiological studies with cells. The enriched view depicts novel targets identified in *C. elegans*, with the exception of NDUSF4, which was found in mouse studies. NALCN+ associated proteins refers to the Na^+^ leak-current channel (NCA-1 and NCA-2 in *C. elegans*) and proteins (UNC-79 and UNC-80) that regulate the expression/function of NALCN. CLICs are chloride intracellular channels. CRF stands for corticotropin-releasing factor, and NPY for neuropeptide Y. While direct binding has not been established in all cases, we have highlighted more direct effects with solid arrows and indirect effects with dashed arrows.

Our understanding of how anesthetics and alcohol work at the molecular level has come a long way [compare Paton and Speden (1965) and Kalant (1974) with Chau (2010) and Howard et al. (2011)]. However, the picture is still incomplete, partly because research has largely focused on how these agents affect individual targets (e.g., GABA_A_ receptors) in isolation. We now realize that multiple proteins and other cell elements are affected, and that the final outcome involves complex interactions of the individual components. Although there is some overlap in the biological actions of anesthetics and ethanol (**Figure 4**), these two classes of agents will be discussed separately.

According to the classical view depicted in **Figure 4**, anesthetics act by modulating various LGICs rather than membrane fluidity as previously believed. In general, they directly activate or potentiate the response of inhibitory GABA_A_ and glycine receptors, and inhibit the activation of excitatory LGICs including nAChRs and *N*-methyl-D-aspartate (NMDA) receptors (Krasowski and Harrison, 1999; Antkowiak, 2001; Miller, 2002; Chau, 2010). Different types of anesthetic agents, e.g., intravenous (etomidate) vs. gaseous (xenon) can act through distinct pathways (Nagele et al., 2005; Forman and Miller, 2011). These and other exceptions to a single mode of action point to the involvement of multiple molecular mechanisms.

Drug elucidation studies in *C. elegans* have revealed genetic mutations that cause either hypersensitivity or resistance to anesthetics (Simpson and Johnson, 1996; Morgan et al., 2007), and the results are summarized in **Figure 4**. These mutations affect neuronal function related to neurotransmitter release, postsynaptic responsiveness and/or mitochondrial energetics (e.g., *gas-1*). Loss-of-function mutations in the *unc-79* and *unc-80* genes, which encode novel proteins that are essential for the expression/function of the Na^+^ leak-current channel (NALCN; NCA-1 and NCA-2 in *C. elegans*), cause hypersensitivity to halothane. In contrast, loss-of-function mutations in *unc-7* and *unc-9*, which encode gap junction proteins, and *unc-1* and *unc-24*, which encode stomatin proteins, suppressed this hypersensitivity when introduced into *unc-79* or *unc-80* (Sedensky and Meneely, 1987; Morgan et al., 1990). Together, NCA-1/NCA-2, UNC-79, and UNC-80 augment depolarization of neurons and vesicular release (Humphrey et al., 2007; Yeh et al., 2008). Findings in *C. elegans* confirm that anesthetics also suppress vesicular release by directly binding to syntaxin and/or SNARE complexes (van Swinderen et al., 1999). Finally, anesthetics regulate neurotransmitter release by targeting G proteins and various regulators of G proteins (van Swinderen et al., 2001; Hawasli et al., 2004).

Based on the results of drug elucidation studies, we propose the following scheme to explain the various actions of anesthetic agents. Anesthetics inhibit complex I activity via GAS-1 and associated proteins (Kayser et al., 1999; Falk et al., 2006). This will decrease NAD^+^ leading to a concomitant reduction in cADP ribose and nicotinic acid-adenine dinucleotide phosphate (NAADP), which normally promote Ca^++^ release from internal sites. The decrease in intracellular Ca^++^ reduces neurotransmitter release, but not sufficiently to cause loss of consciousness. Anesthetic binding to syntaxin-SNARE complexes further compromises neurotransmission. In addition, anesthetics bind to SLO-1 BK channels and potentiate anesthetic effects by hyperpolarizing neurons (Hawasli et al., 2004). Loss-of-function mutations in *unc-79* and *nca-1;nca-2* strains would enhance sensitivity to certain anesthetics by decreasing depolarization-mediated Ca^++^ influx. The absence of UNC-7 or UNC-9 reverses hypersensitivity in these strains by reducing functional gap junctions, which blocks the spread of hyperpolarization, and/or prevents the dissipation of positive signals (e.g., Ca^++^ influx and NAD^+^) involved in neurotransmitter release and excitation. Postsynaptic effects of anesthetics on LGICs and voltage-gated ion channels add to the deficits in neurotransmission to produce anesthesia. Loss of sensation and consciousness will depend on cumulative effects of anesthetics on multiple targets. Finally, different anesthetics have distinct modes of action related to the particular mix of primary and secondary molecular targets they affect.

Ketamine is a dissociative anesthetic that, among other effects, non-competitively inhibits NMDA receptors (Krystal et al., 1994; **Figure 4**). Loss-of-function mutations in *unc-68* reduce sensitivity to ketamine and reveal the ryanodine receptor (RyR) as an additional target (Sakube et al., 1997). The RyR controls Ca^++^ release from internal stores, which is consistent with the model presented above. In addition, ketamine targets NDUSF4, an 18 kDa subunit of mitochondrial complex I (Quintana et al., 2012). Ketamine also inhibits the hyperpolarization-activated cation current channel (HCN1), which results in extended hyperpolarization of neurons (Chen et al., 2009). HCN1 knockout mice show a significant decrease in sensitivity to ketamine (Chen et al., 2009).

According to current thinking (classical view depicted in **Figure 4**), ethanol mainly affects the function of voltage-gated ion channels and LGICs (Dopico and Lovinger, 2009; Howard et al., 2011). Drug elucidation studies reveal a much more complex picture (McIntire, 2010). Ethanol overlaps with anesthetics by directly affecting SLO-1 (Davies et al., 2003), UNC-79 (Morgan and Sedensky, 1995), GAS-1 (Morgan and Sedensky, 1995), and proteins that regulate synaptic vesicle release, including UNC-18 (Graham et al., 2009) and RAB-3 (Kapfhamer et al., 2008). Despite these cursory similarities, there are differences, e.g., *unc-79* loss of function causes hypersensitivity to halothane (Sedensky and Meneely, 1987), but resistance to ethanol (Morgan and Sedensky, 1995). Genetic analysis in *Drosophila*, *C. elegans* and mice has revealed that mutations in chloride intracellular channels (CLICs) also modulate responsiveness to ethanol (Bhandari et al., 2012).

Work in *C. elegans* has also implicated neuropeptide Y (NPY) and corticotropin releasing factor (CRF) signaling in the regulation of ethanol responsiveness. More specifically, loss-of-function mutations in the NPY receptor, *npr-1*, accelerate development of acute tolerance to ethanol (Davies et al., 2004), and gain-of-function mutations in the CRF receptor, *seb-3*, likewise enhance recovery from ethanol exposure (Jee et al., 2013). Conversely, *seb-3(lf)* mutants fail to develop acute tolerance to ethanol (Jee et al., 2013). NPR-1 and SEB-3 do not appear to be direct targets; however, they play a significant role in regulating behavioral responses to ethanol. These drug elucidation studies in *C. elegans* have therapeutic implications. For example, Jee et al. (2013) have suggested that CRF_1_ receptor antagonists might be useful in the treatment of alcoholism.

## OTHER CNS DRUGS IN CLINICAL USE: LITHIUM AND RILUZOLE

Lithium is a front-line treatment for bipolar disorder (Malhi et al., 2012). Although it is perhaps the simplest CNS drug of all in terms of structure, being an element, not a molecule, lithium’s actions are diverse, and its therapeutic mechanisms are not fully characterized. Classically, lithium is considered to work by affecting neurotransmitter release, monoamine metabolism and neuronal excitability by directly targeting G proteins, myo-inositol monophosphatase (IMP) and glycogen synthase kinase-3α (GSK-3α; Phiel and Klein, 2001; Can et al., 2014). Research on *Drosophila* has identified the Wnt signaling pathway as a target of lithium (Berger et al., 2005). Recent findings in *C. elegans* have also advanced our understanding of how this drug may affect the CNS. Weeks et al. (2011) discovered that 5 mM lithium activates SGK-1 via a signaling pathway that includes G proteins and β-arrestin. This leads to phosphorylation of the FOXO protein, DAF-16, and its exclusion from the nucleus. Although 5 mM lithium is somewhat higher than the serum therapeutic range in humans (∼1 mM), in *C. elegans* a cuticle barrier limits uptake of drug, so *in vivo* concentrations are substantially less than the concentration on the culture plate (McColl et al., 2008). Activation of the SGK-1 pathway is neuroprotective (Schoenebeck et al., 2005), which may account for some of the beneficial clinical effects of lithium. Moreover, lithium’s effects on SGK-1, a regulator of extracellular fluid volume and sodium homeostasis (Chen et al., 1999), may explain why this drug can induce diabetes insipidus (Bendz and Aurell, 1999) and hypertension (Vestergaard et al., 1980).

Intriguingly, lithium extends lifespan in *C. elegans* via mechanisms independent of its actions on insulin signaling/DAF-16 (McColl et al., 2008). In these experiments, animals are chronically exposed to 10 mM lithium on plates, with *in vivo* concentrations estimated to reach 1.2 mM. Genomics studies suggest that changes in histone methylation and chromatin structure mediate the increase in longevity. Whether similar changes in brain contribute to the therapeutic actions of lithium in bipolar disorder is a matter of speculation, and a topic for further study.

Riluzole is the only drug currently approved for the treatment of ALS (Gordon et al., 2013). It is thought to spare motor neuron function by decreasing excitotoxicity via direct effects on glutamate release, glutamatergic signaling, and Na^+^ channel inactivation (Doble, 1996). Recent drug elucidation studies of riluzole in *C. elegans* reveal additional targets. Riluzole causes rapid flaccid paralysis of wild-type, young adult *C. elegans* (Dwyer and Aamodt, unpublished observations). Strains harboring double (*avr-14;avr-15*) or triple (*avr-14;avr-15;glc-1*) loss-of-function mutations in glutamate-activated chloride channels are significantly resistant to the effects of riluzole. These *C. elegans* LGICs are most homologous to human glycine receptors (GLRA1-3) that have been implicated in excessive startle syndromes (hyperekplexia) in man (Davies et al., 2010). The double- and triple-mutant strains are also resistant to ivermectin, a nematocidal drug that is an allosteric activator of glutamate-activated chloride channels leading to extended hyperpolarization and paralysis (Dent et al., 2000). Taken together, these studies in *C. elegans* suggest exciting new directions for research in several areas: (1) glycine receptors may be additional targets of riluzole (Mohammadi et al., 2001) and play a role in the pathogenesis of ALS, (2) riluzole may be beneficial in the treatment of hyperekplexia, and (3) riluzole might serve as a lead compound for the development of new anthelmintic agents that overcome emerging drug resistance to ivermectin and related drugs.

## DRUG ELUCIDATION DRIVES DISCOVERY RESEARCH

**FIGURE 5:**
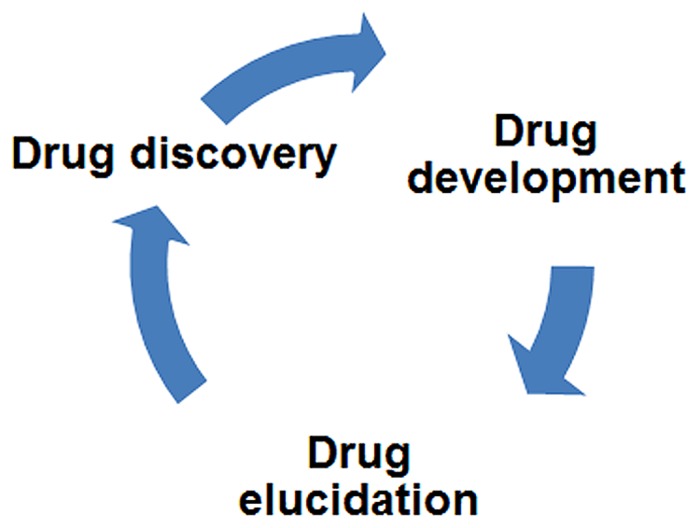
**Drug elucidation can drive drug discovery.** Drug research activities have been depicted in a connected circle to highlight how iterative cycles may operate. Accordingly, drug elucidation is both downstream of drug development (i.e., it focuses on FDA-approved medications) and upstream of drug discovery – identification of novel targets suggests new directions for discovery research. By repeating this cycle with medications already shown to be effective, new drugs can be discovered with fewer side effects and a more rational mode of action.

In this review, we have provided numerous examples where drug elucidation in model organisms led to the identification of novel, unexpected targets of CNS drugs. Beyond these discoveries, drug elucidation studies potentially impact three major areas of research: (1) the development of next-generation drugs, (2) minimization of drug side effects by the avoidance of certain targets in future drugs, and (3) the fundamental understanding of disease mechanisms. Although we picture drug elucidation as a stage that follows drug development, i.e., characterization of drugs already in clinical use, it may also guide new drug discovery as part of an iterative cycle (**Figure 5**). Novel targets identified through drug elucidation become new focal points for the next round of drug discovery. Trace amine receptors in schizophrenia and glycine receptors in ALS are two examples of this prospective approach.

Drug elucidation studies have already provided insights into the side effects of CNS drugs. For example, clozapine and lithium affect SGK-1 signaling, which may explain why these drugs are associated with induction of diabetes insipidus and elevated blood pressure (Weeks et al., 2011). Similarly, ketamine’s effects on the mitochondrial protein NDUSF4 or the RyR may contribute to its reported neurotoxicity (Olney et al., 1989; Scallet et al., 2004). The challenge will be to tease apart the mechanisms causing side effects from those producing therapeutic benefits. Model organisms can be useful in this effort as already demonstrated in *C. elegans* (Donohoe et al., 2006; Karmacharya et al., 2009).

Identification of new targets of approved medications via drug elucidation also expands our knowledge about the pathogenesis of the corresponding diseases. For example, the independent discovery by two groups that APDs activate ISP-Akt signaling in *C. elegans* (Karmacharya et al., 2009; Weeks et al., 2010) is consistent with other evidence suggesting a role for this pathway in schizophrenia. Akt was previously implicated in schizophrenia because single nucleotide polymorphisms in the AKT1 gene were associated with increased risk for this disease, and levels of Akt-1 were reduced in the brains and peripheral blood lymphocytes of schizophrenic patients (Emamian et al., 2004). The prospect of Akt as an attractive therapeutic target in schizophrenia has been discussed in detail elsewhere (Kalkman, 2006; Dwyer and Dickson, 2007). Moreover, the effect of APDs on DAF-16 is especially interesting in view of the fact that this transcription factor regulates the expression of tyrosine hydroxylase (Ferri et al., 2007) and tryptophan hydroxylase (Estevez et al., 2006), and thus the production of dopamine and serotonin, respectively. Importantly, this observation provides a link between the genetics and neurochemistry of schizophrenia and its associated impairment of neuronal function [Akt mediates neuron growth (Dudek et al., 1997; Philpott et al., 1997), soma size (Kumar et al., 2005) and regulates the caliber of neuronal processes (Markus et al., 2002)]. The discovery of trace amine receptors (Karmacharya et al., 2011) and the α7-nicotinic receptor (Saur et al., 2013) as targets of APDs in *C. elegans* spotlight these pathways as candidates for involvement in the pathogenesis of schizophrenia. A role of nicotinic cholinergic systems in schizophrenia has long been suspected based on the high rates of smoking in schizophrenic patients (Freedman et al., 1994).

Similarly, drug elucidation studies of medications used clinically (ADs) or experimentally (ketamine) to treat depressed patients reveal additional signaling mechanisms that should be factored into models about causes of major depression. For example, it is noteworthy that human RyR genes, RYR1 and RYR3, are encoded at sites, 19q13 and 15q14–15, respectively, which have been implicated as risk loci for bipolar disorder in genome-wide association studies (Reif et al., 2004; Francks et al., 2010; Green et al., 2013). Alternatively, these targets may be involved in side effects of ADs such as ventricular tachycardia (Thanacoody and Thomas, 2005; Zima et al., 2008). As discussed here, drug elucidation studies can generate new ideas about disease causation and the adverse effects of psychotropic drugs, and ultimately provide a deeper understanding of CNS disorders. Research on drug effects in model organisms is an essential ingredient of these efforts because genetic manipulations in these systems, especially *C. elegans*, are typically more powerful tools than in mammalian models.

## Conflict of Interest Statement

The authors declare that the research was conducted in the absence of any commercial or financial relationships that could be construed as a potential conflict of interest.
